# Pre-conception blood pressure and evidence of placental malperfusion

**DOI:** 10.1186/s12884-019-2699-3

**Published:** 2020-01-08

**Authors:** Jacqueline Atlass, Marie Menke, W. Tony Parks, Janet M. Catov

**Affiliations:** 1 Department of Obstetrics, Gynecology and Reproductive Sciences, University of Pittsburgh School of Medicine, Pittsburgh, PA USA; 2 Magee-Womens Research Institute, 204 Craft Avenue, Suite A208, Pittsburgh, PA 15213 USA; 3 Department of Pathology, University of Pittsburgh, Magee-Womens Hospital of UPMC, 300 Halket Street, Pittsburgh, PA USA; 4 Present Address: Laboratory Medicine and Pathobiology, University of Toronto, Toronto, ON Canada; 5 Department of Epidemiology, University of Pittsburgh Graduate School of Public Health, Pittsburgh, PA USA

**Keywords:** Placenta, Hypertension, Electronic medical record

## Abstract

**Background:**

Evidence of placental maternal vascular malperfusion is associated with significant perinatal outcomes such as preeclampsia, intrauterine growth restriction and preterm birth. Elevations in pre-pregnancy blood pressure increase the risk for poor perinatal outcomes; however, the evidence linking pre-pregnancy blood pressure and placental malperfusion is sparse.

**Materials and methods:**

We conducted a retrospective case-control study of women with singleton gestations with placental evaluations who delivered at Magee-Womens Hospital in 2012. Charts from 100 deliveries with placental malperfusion lesions (vasculopathy, advanced villous maturation, infarct, or fibrin deposition) and 102 deliveries without placental malperfusion were randomly selected for screening. Blood pressure, demographic, and clinical data were abstracted from pre-pregnancy electronic medical records and compared between women with and without subsequent placental malperfusion lesions.

**Results:**

Overall, 48% of women had pre-pregnancy records, and these were similarly available for women with and without placental malperfusion. Women with placental malperfusion demonstrated a reduction in their pre- to early pregnancy decrease in diastolic blood pressure (DBP). Adjusted for race, pre-pregnancy BMI, age, pre-conception interval, and gestational age at the first prenatal visit, the difference in pre- to early pregnancy DBP was significantly less in women with placental malperfusion compared to those without this pathologic finding (− 1.35 mmHg drop vs − 5.6mmg, *p* < 0.05).

**Conclusion:**

A blunted early gestation drop in DBP may be a risk factor for placental malperfusion, perhaps related to early pregnancy vascular maladaptation. The ability of the electronic medical record to provide pre-pregnancy data serves as an underutilized approach to study pre-pregnancy health.

## Background

Classically, maternal blood pressure decreases very early in the first trimester due to decreased systemic vascular resistance, reaching a nadir at mid-pregnancy after which blood pressure slowly rises to pre-pregnancy levels by term [1–4]. Women with chronic hypertension are at increased risk of developing preeclampsia, fetal growth restriction, placental abruption and preterm delivery, suggesting that pre-pregnancy blood pressure contributes to these adverse outcomes [5–10].

The placenta provides both nutrients and oxygen to a growing fetus, and impairments in early vascularization are related to adverse pregnancy outcomes [11–15]. During the 4th–5th week of placental development, extravillous trophoblasts invade into the endometrium leading to remodeling of the vital spiral arteries. This evolution of the uteroplacental circulation creates a vascular network composed of low resistance vessels. Failure of this critical process underlies many complications and adverse events in pregnancy. Chronic uteroplacental insufficiency creates a state of oxidative stress and injury, contributing to the development of malperfusion lesions [16].

The contribution of pre-pregnancy cardiometabolic risk factors such as blood pressure to placental vascular health is not well understood, as they are not typically available to the obstetrician. We considered that these may be documented in electronic health records, and may be related to the occurrence of maternal vascular malperfusion detected in the placenta. Specifically, maternal pre-pregnancy blood pressure may be an important and under-utilized vital sign.

As a proof of concept, we sought to determine the availability of pre-pregnancy blood pressure measures in the electronic health record, and related these to the occurrence of placental malperfusion. We hypothesized that most women would have pre-pregnancy records, and that higher pre-pregnancy blood pressure would be associated with higher risk for placental malperfusion. We also considered that the well-established early pregnancy drop in blood pressure would reflect uteroplacental function as measured through placental malperfusion lesions.

## Materials and methods

A retrospective case-control study was performed using a clinical cohort of women with singleton gestations who delivered at Magee-Womens Hospital. All deliveries at our institution are registered in the Magee Obstetric Medical and Infant database (MOMI) which includes clinical and demographic features abstracted from the prenatal and delivery medical records. For deliveries occurring in 2008–2012, we additionally abstracted placental features in the subset of births that included a placental evaluation (*n* = 20,012) [17]. Placental evaluations were performed by two perinatal pathologists according to current guidelines [18]. For this pilot study, we selected 200 cases for review based on feasibility of manual chart abstraction. We randomly selected women with births in 2012 according to presence of placental malperfusion lesions (vasculopathy, advanced villous maturation, infarct, or fibrin deposition, *n* = 100) and deliveries without placental malperfusion (*n* = 102) for screening. Criteria for these lesions followed established guidelines, and are summarized in Additional file 1: Table S1 [18]. Women were excluded if pre-pregnancy visit data were unavailable in the electronic medical record (EMR) within 3 years of delivery and prior to the estimated date of conception, based on gestational age at delivery (*n* = 106). We selected 3 years as a reasonable interval to examine the availability of health records in a population for whom annual check-ups are not recommended. Data from cohorts such as the Coronary Artery Risk Development in Young Adults (CARDIA) which are designed to evaluate progression of risk factors such as blood pressure, track data every 5 years due to the evidence that risk factors do not change much in that interval [19].. The pre-pregnancy records preferentially came from an outpatient routine gynecology or primary care exam. If unavailable, the clinical data were extracted from an outpatient acute care visit, and lastly, from an Emergency Department visit. Blood pressure, demographic, and clinical data (height, weight, and medications) were abstracted.

Early pregnancy data were abstracted from the initial prenatal visit. Visits for a positive pregnancy test or a missed menses nurse visit were not used. Pregnancy outcomes including preeclampsia, small for gestational age (SGA, birth weight evaluated according to a referent of estimated fetal weight for gestational age, adjusted for race) [20], and preterm birth were abstracted from delivery records.

Maternal characteristics were compared based on availability of pre-pregnancy records and presence of placental malperfusion lesions using t-tests or chi-squared tests. We used linear regression to estimate the systolic and diastolic blood pressures both pre-pregnancy and early gestation, and the mean change in blood pressure between these time points according to presence of malperfusion lesions. Covariates selected a priori included age, race, pre-pregnancy BMI, pre-pregnancy interval (estimated date of conception - date of EMR visit) and estimated gestational age at first prenatal visit. Analyses were replicated in the group with routine office visit charts. We adhered to STROBE guidelines and methodology.

## Results

Of the 202 charts reviewed, 96 women had pre-pregnancy visits in the EMR (48%). Women with pre-pregnancy records had higher rates of smoking compared to those without available records. There were no other differences in maternal characteristics according to availability of pre-pregnancy charts [Table 1]. Among those with pre-pregnancy records, women with placental malperfusion lesions (*n* = 49) were more likely to be white (85% vs 65%, *p* = 0.04) and tended to have lower pre-pregnancy BMI (29.4 + 14.7 vs 32.3 + 16.8, *p* = 0.06, Table 2) compared to women with no malperfusion (*n* = 47). As expected, women who developed preeclampsia or delivered preterm infants were more likely to have placental malperfusion, although these differences were not statistically significant. Most pre-pregnancy blood pressure measures were obtained from routine office visits, regardless of the subsequent occurrence of placental malperfusion.

**Table 1:** Maternal characteristics according to availability of pre-pregnancy charts

	No pre-pregnancy charts	Pre-pregnancy charts	p
	*n* = 106	*n* = 96	
Maternal age			0.20
< 20	6 (6)	6 (6)	
20–29	53 (50)	47 (49)	
30 + −39	47 (44)	43 (45)	
Race/ethnicity			
White	79 (75)	70 (73)	0.07
Black	16 (15)	23 (24)	
Other	11 (10)	3 (3)	
Smoking	7 (7)	14 (15)	0.04
Education^a^			0.97
High school	28 (30)	25 (34)	
College	47 (51)	34 (47)	
College+	17 (19)	14 (19)	
Hypertension			0.73
None	76 (72)	73 (76)	
Gestational	11 (10)	11 (12)	
Preeclampsia	17 (16)	10 (10)	
Chronic	2 (2)	2 (2)	
Diabetes^a^			0.41
None	94 (89)	84 (90)	
Gestational	10 (9)	9 (10)	
Pre-existing DM	2 (2)	0	
Primiparous	75 (71)	55 (57)	0.09
Fetal sex male	52 (49)	51 (53)	0.31
Gestational age, weeks	37.6 (3.2)	38.0 (3.1)	0.30
Preterm birth			0.14
Term	82 (77)	84 (88)	
Spontaneous	12 (11)	8 (8)	
Indicated	12 (11)	4 (4)	

^a^Missing data for the following, Education: *n* = 14 for women with no pre-pregnancy charts; *n* = 23 for women with pre-pregnancy charts; Diabetes: *n* = 3 for women with pre-pregnancy records

**Table 2:** Maternal characteristics of those with pre-pregnancy charts, according to presence of malperfusion lesions

	No Malperfusion	Malperfusion	
	*n* = 47	*n* = 49	p
Reason for pre-pregnancy visit			0.23
Office/annual	32 (68)	29 (59)	
Problem, office visit	10 (21)	17 (35)	
Emergency room	5 (11)	3 (6)	
Age			0.30
< 20	3 (6)	3 (6)	
20–29	24 (51)	24 (49)	
30 +	20 (43)	22 (45)	
Smoking	8 (17)	6 (12)	0.67
Race			
White	31 (67)	40 (82)	0.19
Black	15 (31)	9 (18)	
Other	1 (2)	0	
Pre-pregnancy BMI, k/m^2^	30.9 (11)	27.2 (6)	0.06
Hypertension			0.27
Gestational	8 (17)	3 (6)	
Preeclampsia	3 (6)	7 (14)	
Preterm birth	4 (8)	8 (16)	0.13
Gestational diabetes	3 (6)	6 (12)	0.32
Small for gestational age	7 (15)	8 (16)	0.92
Malperfusion lesions, n (%)^a^			
Vasculopathy		8 (16)	
Syncytial knots		8 (16)	
Infarct		24 (49)	
Fibrin deposition		14 (29)	
Pre-pregnancy Medication use			
Antibiotics	3 (6)	6 (12)	0.32
Asthma	5 (11)	4 (8)	0.68
Antihypertensive	3 (6)	0	0.07
Antidiabetic	1 (2)	1 (2)	0.98
Anxiety	2 (4)	1 (2)	0.53
Antidepression	6 (13)	4 (8)	0.46
Lipid lowering	2 (4)	3 (6)	0.68

^a^Women may have more than one malperfusion lesion detected in the placenta

Gestational age at first prenatal visit did not differ based on presence of placental malperfusion. Amongst women with and without placental malperfusion, there were no differences in pre-pregnancy systolic (SBP, 114.3 + 10.6 vs 117.3 + 12.6 mmHg, *p* = 0.22) or diastolic blood pressure (DBP, 71.7 + 8.2 vs 74.3 + 10.1 mmHg, *p* = 0.18; Table 3). Similarly, early pregnancy systolic (*p* = 0.35) and diastolic blood pressures (*p* = 0.64) were not associated with placental malperfusion.

**Table 3:** Systolic and diastolic blood pressure before and during early pregnancy, according to presence of placental malperfusion lesions, mean and SD

	No Malperfusion	Malperfusion	
	*n* = 47	*n* = 49	p
SBP, pre-pregnancy	117.3 (12.6)	114.3 (10.6)	0.22
SBP, pregnancy	115.6 (10.6)	111.7 (9.3)	0.35
SBP difference (prepregnancy-pregnancy)	2.2 (15.4)	1.9 (15.7)	0.93
DBP, pre-pregnancy	74.3 (10.1)	71.7 (8.2)	0.18
DBP, pregnancy	69.3 (8.3)	70.1 (7.3)	0.60
DBP difference (prepregnancy-pregnancy)	5.0 (11.9)	1.6 (10.1)	0.15
GA at prenatal visit, weeks (95% CI)	10.0 (8.8, 11.2)	9.4 (8.1, 10.7)	0.52
Preconception interval, months (95% CI)	7.9 (5.7, 10.1)	10.0 (7.3, 12.7)	0.22

**Fig. 1:**
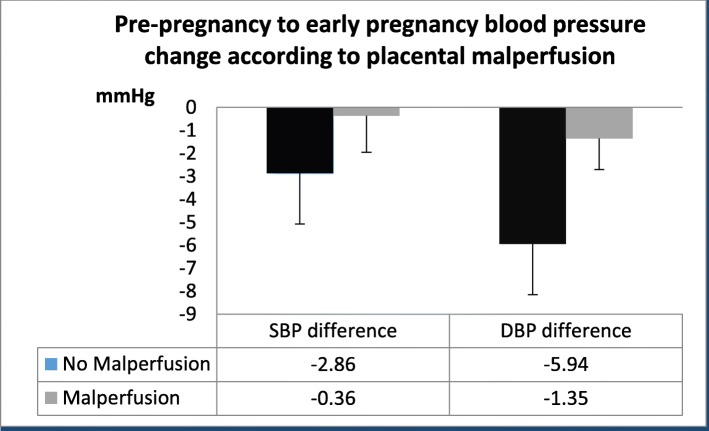
Comparing the diastolic blood pressure difference in a pre-pregnancy versus early pregnancy state according to placental malperfusion

The change from pre-pregnancy to early pregnancy DBP was not different according to presence of placental malperfusion lesions (− 1.6 + 10.1 vs − 5.0 + 11.9 mmHg, *p* = 0.15). After accounting for race, pre-pregnancy BMI, age, pre-conception interval, and gestational age at the first prenatal visit, however, the DBP difference was significantly less in women with placental malperfusion lesions compared to those without this pathologic finding (− 1.35 mmHg vs − 5.6mmgHg, *p* = 0.048; Fig. 1). There was a smaller pre- to early pregnancy drop in SBP among those with placental malperfusion lesions, but this was not statistically significant (− 0.36 vs. -2.86 mmHg, *p* = 0.437 adjusted for covariates). Results were similar when analysis was restricted to those for whom blood pressure measures were collected from routine office visits (*n* = 61) where the DBP drop was blunted in those with subsequent placental malperfusion compared to no malperfusion (− 0.81 vs. -4.76 mmHg, *p* = 0.066).

## Discussion

Our findings reveal that women with placental malperfusion lesions may have a blunted pre- versus early pregnancy decrease in diastolic blood pressure. Although reasons for placental malperfusion are not well understood, it is possible that abnormal placental development alters maternal blood pressure changes. Alternatively, abnormal blood pressure changes may be evidence of a pre-existing maternal vascular phenotype that is more susceptible to vascular impairments detected in the placenta.

Physiologic decreases in maternal blood pressure in early pregnancy have been well-described, and are explained by hemodynamic changes within the maternal vasculature. Page and Christianson first described a “classic” mid-trimester drop in blood pressure which returns to baseline and even rises towards term [11]. Arterial blood pressure has been found to decrease as early as the 7th week of gestation. While systolic BP tends to remain relatively stable throughout pregnancy, diastolic BP typically nadirs during the 2nd trimester, most commonly between 16 and 24 weeks. DBP tends to follow a “J shaped pattern” across the trimesters, but little is known regarding the pre-pregnancy to early BP patterns [2–4, 21–27]. Most BPs reported in pregnancy studies are measured beginning around 8 weeks’ gestation; thus, the association between pre- and early pregnancy blood pressure remains unclear. However, consistent with our results, a prior report of maternal blood pressure measured up to 2 years prior to conception also demonstrated brachial SBP and DBP reductions by 6 to 7 weeks’ gestation (− 4 mmHg SBP, − 6 mmHg, respectively) in uncomplicated pregnancies [28]. As expected, these were accompanied by drops in peripheral vascular resistance, consistent with the possibility that a blunted BP drop may be related to impairments in vascular adaptations detectable at delivery through examination of the placenta.

As expected, women who developed preeclampsia or delivered preterm infants were more likely to have placental malperfusion lesions in our study. Importantly, not all women with evidence of placental malperfusion developed these complications, raising the possibility that these maternal vascular lesions may be related to an occult vascular phenotype at minimum or worse, indicate adverse subsequent pregnancy health and/or long term maternal cardiovascular disease. These possibilities warrant future study.

Maternal blood pressure is a critical vital sign documented at each prenatal visit in the electronic medical record. There is little information regarding the use of pre-pregnancy EMRs in prenatal care. One study in family medicine found that using an EMR increased the likelihood that patients had screening tests performed on time, suggesting that the availability of EMRs may contribute to improved obstetrical care [29]. In an era of ‘big data’ there is an increased availability of pre-pregnancy health data. Our study demonstrates how the EMR within an integrated health system may enhance risk identification during pregnancy. The ability of the EMR to provide pre-pregnancy data serves as an untapped opportunity to study pre-pregnancy health. Identification of women with a blunted DBP drop at the first prenatal visit, for example, may prompt closer maternal and fetal monitoring to decrease morbidity.

An important limitation of our study is that only half of women had pre-pregnancy records available. This limitation is likely one of the population studied. For young, healthy women, the prenatal visit may be their first medical care encounter and their first measured blood pressure within the EMR. For almost half of women, however, there may be pre-pregnancy health information, including blood pressure, that could provide valuable data to be included in a low-cost risk stratification algorithm. As a pilot study, our report demonstrates the utility and the feasibility of gathering pre-pregnancy clinical data from the EMR, although these results would require replication in a larger cohort and include other institutions to ensure generalizability. We relied on clinical BP measures, which are known to vary. There is evidence, however, that clinical measures are not systematically different from research BP measures [30] and many large studies have relied on clinical BP measures to describe patterns associated with adverse pregnancy outcomes [31]. In addition, larger studies with a greater number of non-acute pre-pregnancy BP measures are needed to validate our findings.

## Conclusions

Our study highlights the importance of pre-pregnancy cardiometabolic risk factors and their potential influence on pregnancy and placental health. Of note, about half of women had pre-pregnancy records available. This number is large, but not complete and it is possible that this proportion may increase as the use of the electronic medical record in health systems continues to expand. While much is known about the classic mid-trimester drop in blood pressure, our study emphasizes the potential importance of pre- to early blood pressure change. Women with placental evidence of malperfusion injury may have a blunted very early DBP drop. Despite the limitations of clinical blood pressure measurements, we detect what may be an important precursor to poor placental vascular health.

## Supplementary information

**Additional file 1: Table S1**. Diagnostic criteria for placental lesions.

## Data Availability

The datasets used and/or analysed during the current study are available from the corresponding author on reasonable request.

## Abbreviations

BMI: Body-mass index
DBP: Diastolic blood pressure
EMR: Electronic medical record
MOMI: Magee Obstetric Medical and Infant Database
SBP: Systolic blood pressure
SGA: Small for gestational age
