# Supplementary material for: Pre-conception blood pressure and evidence of placental malperfusion
Source: BMC Pregnancy Childbirth. 2020 Jan 8;20:25. doi: 10.1186/s12884-019-2699-3 (PMC6950980; doi:10.1186/s12884-019-2699-3)
Supplement: Supplementary file 1 — Additional file 1: Table S1. Diagnostic criteria for placental lesions. [file 12884_2019_2699_MOESM1_ESM.docx]

**Table S1. Diagnostic criteria for placental lesions**

| Placental lesion | Diagnostic criteria |
| --- | --- |
|  |  |
| Acute chorioamnionitis | A maternal response to infected amniotic fluid characterized by the presence of a linear accumulation of neutrophils within the subchorionic fibrin or the chorionic plate itself. |
| Acute vasculitis | A fetal response to infected amniotic fluid characterized by neutrophils within or emerging from the vessels of the fetal chorionic plate or umbilical cord. |
| Acute funisitis | Acute vasculitis of the umbilical cord vessels in which the neutrophils traverse through the vessel wall into the surrounding Wharton’s jelly. |
| Acute deciduitis | A significant linear accumulation of neutrophils within the decidual tissues of the placental basal plate or the extraplacental membranes. |
|  |  |
| Decidual vasculopathy | Incomplete, pathologically abnormal remodeling of maternal vessels supplying the placenta, with four often co-occurring manifestations. Absence of vascular remodeling is defined by the presence of a smooth muscle wall in at least one decidual vessel of the placental basal plate. Mural hypertrophy of decidual arterioles is characterized by thickening of the muscle wall of a decidual vessel from any location, with the thickened muscle wall leaving a luminal diameter of less than 30% of the total vessel diameter. Fibrinoid necrosis of vessel walls presents as a waxy, intense red degeneration of at least one decidual vessel wall from any location. Atherosis is defined by the presence of foamy macrophages within at least one decidual vessel wall. Fibrinoid necrosis and atherosis commonly co-occur. |
| Villous infarction | Devitalization of a region of placental villi due to obstruction of the underlying maternal blood flow. Characterized by a geographically limited loss of staining, often with collapse of the intervening maternal blood space. |
| Advanced (accelerated) villous maturation | Characterized by the presence of at least two specific pathologic changes in the villous architecture. Advanced villous maturation is typically characterized by an increase in the percentage of villi containing a syncytial knot (increased syncytial knots), a decrease in the percentage of intermediate villi and/or distal villous hypoplasia (zones of abnormally long, thin, unbranched terminal villi). |
| Perivillous fibrin deposition | Irregular zones of fibrinoid material tightly encasing the entrapped villi. A small amount of perivillous fibrin is acceptable in the upper third of the placental parenchyma. |
| Intervillous fibrin deposition | An increased percentage (3% is the upper limit) of small foci of fibrinoid material within or adjacent to villi. |
